# Italiens Bankensystem und die Coronakrise: Risiken durch die enge Verflechtung zwischen Staaten und Banken

**DOI:** 10.1007/s10273-022-3116-5

**Published:** 2022-02-19

**Authors:** Jürgen Matthes

**Affiliations:** Kompetenzfeldes Internationale Wirtschaftsordnung und Konjunktur, Institut der deutschen Wirtschaft Köln e.V., Konrad-Adenauer-Ufer 21, 50668 Köln, Deutschland

**Keywords:** G2, O5, E6

## Abstract

Das italienische Bankensystem ist bislang recht gut durch die Coronakrise gekommen. Dazu haben umfangreiche staatliche Hilfsmaßnahmen ebenso beigetragen wie Vorkrisenreformen mit Blick auf Altlasten aus der Euro-Schuldenkrise. Dazu zählen der Abbau notleidender Kredite und der Aufbau von Kapitalpuffern. Doch 2022 könnten Firmeninsolvenzen zur erneuten Belastungsprobe werden, wenn staatliche Kreditgarantien und -moratorien allmählich auslaufen. Risiken liegen für das italienische Bankensystem mittelfristig vor allem in der engen Verflechtung zwischen Staat und Banken. Eine Staatsschuldenkrise droht damit rasch zu einer umfassenden Wirtschaftskrise zu werden.

## Abstract (en)

The Italian banking system has come through the coronavirus crisis quite well so far. Extensive government aid measures have contributed to this as well as pre-crisis reforms intended to eliminate legacy burdens from the euro debt crisis. These include the reduction of non-performing loans and the build-up of capital buffers. But in 2022, corporate insolvencies and problems with private customers could become a new test of endurance when state credit guarantees and moratoria gradually expire. Risks for the Italian banking system in the medium term are mainly due to the close ties between the state and the banks. A looming sovereign debt crisis thus threatens to quickly turn into a comprehensive economic crisis.

Die Entwicklung des Abbaus der Altlasten und zukünftiger Risiken im italienischen Bankensystem hat eine hohe wirtschaftliche und politische Bedeutung in der Europäischen Union (EU). So entscheidet die Resilienz der italienischen Banken und ihre Kreditvergabefähigkeit in und nach der Coronakrise mit darüber, ob die europäischen Aufbaufinanzen und die Reformen der Regierung Draghi das chronisch schwache Wachstum in Italien stärken können. Das ist wichtig, um die hohe Staatsverschuldung in Italien tragfähig zu halten. Belastungsfaktoren für die italienischen Banken sind neben der Coronakrise, die die heimische Wirtschaft hart traf, auch noch verbliebene Altlasten aus der Euro-Schuldenkrise. Diese Frage ist auch für weitere Integrations- und Solidaritätsschritte in der Europäischen Währungsunion (EWU) relevant. Das gilt etwa für die Vollendung der Bankenunion, die sowohl im Koalitionsvertrag der deutschen Bundesregierung (SPD et al., 2021) als auch im Programm der französischen EU-Ratspräsidentschaft (Französischer Vorsitz im Rat der EU, 2022) als Ziel genannt wird. Hier geht es um die Frage, ob, unter welchen Bedingungen und in welcher Form eine gemeinsame Einlagensicherung für die EWU-Banken eingerichtet werden kann. Weil damit eine Vergemeinschaftung von Risiken verbunden wäre, war politisch vereinbart, dass zuvor die Altlasten der Euro-Schuldenkrise hinreichend abgebaut und neue Risikopuffer aufgebaut werden.

### Italiens Bankensystem bis zur Krise

Italiens Bankensystem wurde während der Euro-Schuldenkrise stark gebeutelt. Die Wirtschaftskrise schwächte Unternehmen und deren Fähigkeit, Kredite zu bedienen, der Anteil notleidender Kredite schnellte in die Höhe und aufgrund von Verlusten schmolz die Kapitalbasis der Banken. Zudem gerieten die italienischen Banken von zwei weiteren Seiten unter Druck. Zum einen zogen Banken anderer Länder und internationale Investierende kurzfristige Kredite im Interbankenmarkt und darüber hinaus zurück, sodass auch die Liquiditätsversorgung der Banken litt und die EZB einspringen musste. Zum anderen gerieten die italienischen Banken in einen Teufelskreis mit dem hoch verschuldeten italienischen Staat. Beide hingen so voneinander ab, dass sie sich gegenseitig in eine immer tiefere Krise zu ziehen drohten.

Es dauerte lange, bis wichtige Altlasten der Euro-Schuldenkrise entschieden in Angriff genommen wurden. Erst spät ging man das Problem der notleidenden Kredite an, dann aber – auch durch Drängen der Euro-Partner – mit Tempo und Durchschlagskraft. Der Anteil notleidender Kredite an allen Krediten sank in Italien von immensen 17 % Mitte 2015 auf 6,7 % Ende 2019 vor der Coronakrise (vgl. Abbildung 1). Die italienischen Banken gewannen dabei wichtige Erfahrungen mit Instrumenten zur Veräußerung notleidender Kredite. Trotz des enormen Fortschritts stand Italien immer noch schlechter da als viele andere Euroländer. Der EWU-Durchschnitt betrug Ende 2019 3,2 % (vgl. Abbildung 2). Diese und die direkt folgenden

**Jürgen Matthes** leitet das Kompetenzfeld Internationale Wirtschaftsordnung und Konjunktur am Institut der deutschen Wirtschaft.

Abbildung 1
**Entwicklung notleidender Kredite bei Italiens Banken**
Anteil notleidender Kredite an allen Krediten in %

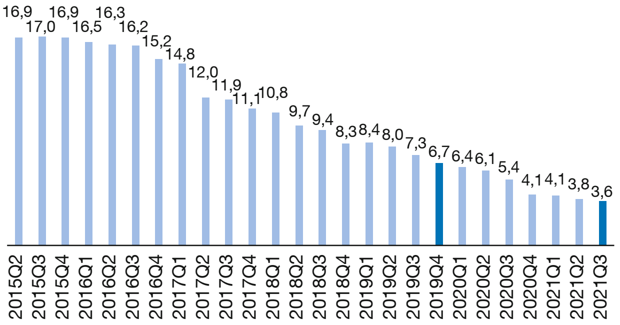

Angaben für signifikante Banken unter EZB-Aufsicht.

Quelle: ECB (2022).

Abbildung 2
**Notleidende Kredite im EWU-Vergleich**
Anteil notleidender Kredite an allen Krediten in % im vierten Quartal 2019 und im dritten Quartal 2021

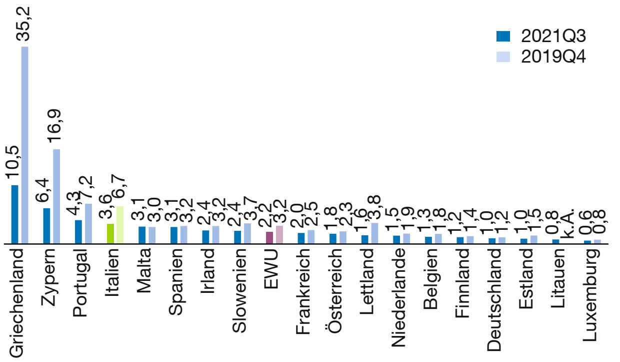

Angaben für signifikante Banken unter EZB-Aufsicht. Keine Angaben für Slowakei und 2019 für Litauen, Ranking gemäß der Werte von 2021.

Quellen: ECB (2022; 2020a; 2021a).

Angaben beziehen sich auf die von der EZB überwachten signifikanten Banken, von denen es in Italien elf gibt. Auch die Kapitalpuffer zur Abfederung künftiger Risiken wuchsen zwischen 2015 und 2019 – gemessen an der harten Kernkapitalquote (CET 1) von 11,5 % auf 14 % der risikogewichteten Aktiva (vgl. Abbildung 3). Damit schnitt Italien im EWU-Vergleich gleichwohl nur mäßig ab, befand sich aber nah am EWU-Durchschnitt, der von den großen Ländern geprägt wird (vgl. Abbildung 4).

Bei dem Nexus zwischen Staaten und Banken – einer weiteren Altlast aus der Euro-Schuldenkrise – ist dagegen nichts passiert, obwohl dieser in der Euro-Schuldenkrise zu einem gefährlichen Teufelskreis führte (Demary und Matthes, 2013). Zwar ging der Anteil der von den italienischen Banken gehaltenen Staatsanleihen zwischen Ende 2015 und Ende 2019 von rund 30 % auf etwa 20 % zurück, was auch mit den Ankaufprogrammen der EZB zu tun haben dürfte. Doch blieb der Anteil der gehaltenen Staatsanleihen an der Bilanzsumme der italienischen Banken, der im Zuge der Euro-Schuldenkrise auf neue Höchststände gestiegen war, im gleichen Zeitraum auf sehr hohem Niveau. Mit 10,3 % verzeichnete Italien Ende Dezember 2019 die höchste Quote unter allen Euroländern. In Deutschland und Frankreich betrug diese weniger als 2 % (vgl. auch Abbildung 7), wobei in Deutschland zu berücksichtigen ist, dass diese Statistik kleinere Banken wie Sparkassen nicht erfasst, die eng mit deutschen Kommunen verflochten sind. In Spanien, Portugal, Griechenland, Zypern liegen die Quoten zwischen knapp 6 % und 8 %. Der Anteil an der Bilanzsumme ist der deutlich wichtigere Indikator, weil er die Verwundbarkeit der Banken aufzeigt, die bei Unsicherheiten über die Tragfähigkeit der Staatsschulden und sich dadurch ergebenden Kursverlusten entstehen würde. Da die Staatsschulden bei Italien besonders hoch sind, ist diese Konstellation sehr bedenklich.

Auch stiegen die Verbindlichkeiten des italienischen Bankensystems im Rahmen des EWU-Zahlungssystems Target2 zwischen 2015 und 2019 weiter – von einer weiten Spanne um die 200 Mrd. Euro auf zwischen 400 Mrd. und 550 Mrd. Euro (Sinn, 2022). Hier spielen ebenfalls die Ankaufprogramme der EZB eine Rolle, unter anderem weil die Banca d'Italia auch in Frankfurt a. M. italienische Staatsanleihen kauft und weil verkaufende internationale Anlegende ihre Erlöse nicht mehr in Italien anlegten. Dies ist zwar ein gewisses Misstrauensvotum gegenüber dem italienischen Bankensystem, sollte aber auch nicht überbewertet werden.

Insgesamt wies das italienische Bankensystem im Winter 2020, als die Coronakrise begann, zwar weiterhin Schwächezeichen auf, war aber durch den starken Abbau der notleidenden Kredite und den weiteren Aufbau der Kapitalpuffer besser aufgestellt als es noch 2015 zu erwarten war. Das zeigte sich auch an einer moderaten Profitabilität von 4,8 % im Jahr 2019 (Return on Equity), die höher lag als etwa in Deutschland mit nur 1,7 % (EBF, 2020).

### Entwicklungen während der Krise

Gleichwohl bestanden am Anfang der Krise große Sorgen, dass die italienischen Banken erneut in eine tiefe Krise geraten könnten. Denn Italien war anfänglich besonders stark von der Coronakrise betroffen. Zudem griff ein früher und besonders strikter Lockdown. Das traf die Wirtschaft durch ihre Branchenstruktur – mit einem relativ hohen Anteil von Tourismus und Gastgewerbe – vergleichsweise hart. Bei massenhaften Unternehmenspleiten und Zahlungsproblemen, so die Sorge, wäre es bei den Banken

Abbildung 3

**Entwicklung der Kapitalpuffer von Italiens Banken**

Anteil von Kernkapital (Tier1 Ratio) und hartem Kernkapital (CET1 Ratio) an den risikogewichteten Aktiva in %

Angaben für signifikante Banken unter EZB-Aufsicht.

Quelle: ECB (2022).

Abbildung 4

**Hartes Kernkapital im EWU-Vergleich**

Anteil von hartem Kernkapital (CET1 Ratio) an den risikogewichteten Aktiva in %

Angaben für signifikante Banken unter EZB-Aufsicht. Keine Angaben für Slowakei und 2019 für Litauen, Ranking gemäß der Werte von 2021.

Quellen: ECB (2022; 2020a; 2021a).

zu starken Kreditausfällen und Verlusten gekommen, sodass die Banken ihre Kreditvergabe in und nach der Krise hätten einschränken müssen und es womöglich zu einer Kreditklemme gekommen wäre. Um das zu verhindern, ergriff der italienische Staat umfangreiche Hilfsmaßnahmen. Dazu zählen vor allem eine zeitweise Aussetzung der Insolvenzregeln, Kreditschuldenmoratorien und staatliche Garantien für Bankkredite. Zudem stellte die EZB Liquidität über zusätzliche Refinanzierungsmöglichkeiten bereit und schuf ein neues krisenbezogenes Ankaufprogramm für Staatsanleihen, auch um die Finanzmärkte zu beruhigen.

Die italienischen Wirtschaftsakteur:innen griffen stark auf viele dieser Hilfsinstrumente zurück. Nach Angaben des IWF (IMF, 2021) profitierten zwischenzeitlich rund 14 % der Bankkredite insgesamt von Moratorien. Nicht finanzielle Firmen nutzen diese Möglichkeit deutlich stärker als private Haushalte. Das gilt auch für staatliche Kreditgarantien. Rund 18 % der Bankkredite an nicht finanzielle Unternehmen waren nach IWF-Angaben ganz oder teilweise mit solchen Garantien versehen. Diese Maßnahmen trugen dazu bei, die Kreditqualität der Banken zu schonen und eine Kreditklemme während der Krise zu verhindern. Die Kreditvergabe entwickelte sich während der Krise deutlich günstiger als in der Zeit nach der Euro-Schuldenkrise und reflektierte den hohen krisenbedingten Liquiditätsbedarf der Privatwirtschaft – der Kreditanstieg ging aber vollständig auf Kredite mit staatlichen Garantien zurück. Solche banken- und kreditbezogenen Sondermaßnahmen wurden in Italien Ende 2020 dreimal stärker genutzt als im EU-Durchschnitt – gemessen am Anteil an den vergebenen Krediten (IMF, 2021). Im vierten Quartal 2020 gingen z. B. über ein Drittel der Kredite mit Moratorien in der EU auf Italien zurück. Auch auf EZB-Sonderliquiditätsmaßnahmen griffen italienische Banken besonders stark zurück.

Abbildung 5 macht deutlich, dass die Hilfsmaßnahmen vor allem bei der Kreditvergabe an Privatkundschaft erhebliche Auswirkungen in Italien gehabt haben dürften. So gingen hier die Ausfallraten sehr deutlich zurück, von 4,8 % Ende 2019 auf 1,8 % im dritten Quartal 2021. Eine nicht ganz so starke Verbesserung zeigt sich bei den Verlustquoten bei der Privatkundschaft. Diese Angaben der Europäischen Bankenaufsichtsbehörde (EBA) gelten für tendenziell größere Banken, die einen internen ratingbasierten Ansatz zur Ermittlung ihrer Kapitalanforderungen nutzen dürfen. Ein wichtiger Unterschied zu Deutschland fällt bei der Privatkundschaft ins Auge. Die Ausfallraten und auch die Verlustquoten liegen hierzulande zu beiden Betrachtungszeitpunkten deutlich niedriger. Auch ist in Deutschland 2021 nur eine unwesentliche Verbesserung zu erkennen. Bei der Kreditvergabe an Unternehmen sind weniger Unterschiede zwischen Italien und Deutschland vorhanden. Doch auch hier fällt ins Auge, dass die Ausfallraten in Italien deutlich gesunken sind. Dies gilt auch, aber weniger stark für Deutschland.

Im Jahresverlauf 2021 verstärkte sich die Divergenz bei der Nutzung staatlicher Hilfen zwischen Italien und der EU noch, auch weil in vielen anderen Ländern Moratorien beendet wurden (etwa in Frankreich und Spanien), während sie in Italien verlängert wurden. Selbst im Herbst 2021 waren nach Angaben der EZB (ECB, 2021b) noch zwischen 5 % und 10 % der neuen Kredite mit staatlichen Garantien versehen. Der starke Rückgriff auf die Hilfen kann als Indiz für gewisse inhärente Schwächen im italienischen Bankensystem gesehen werden. Die staatliche Hilfe trug auch dazu bei, dass sich der Anteil notleidender Kredite weiter verringerte – von 6,7 % Ende 2019 auf 3,6 %

Abbildung 5

**Ausfallraten und Verlustquoten bei Italiens Banken***

in %

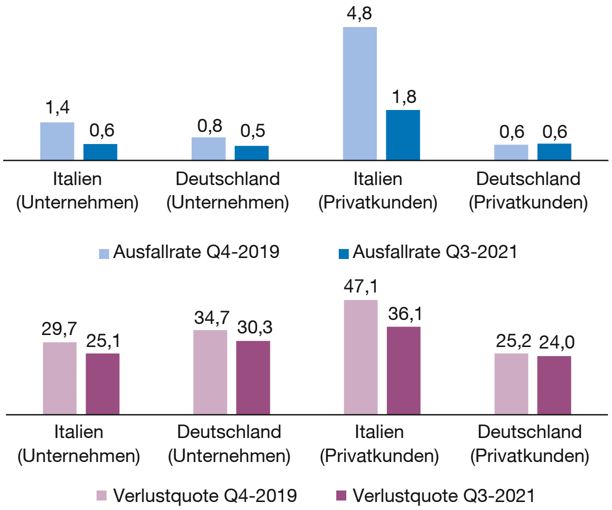

*Nur Banken mit der Erlaubnis für internen ratingbasierten Ansatz zur Ermittlung der Kapitalanforderungen. Kreditvergabe an Privatkundschaft schließt Immobilienkredite ein.

Quellen: EBA (2019; 2021).

im dritten Quartal 2021 (vgl. Abbildung 1). Damit schneidet Italien aber weiterhin etwas schlechter ab als die meisten anderen Euroländer (EWU-Durchschnitt 2,2 %). Auch die Kapitalpuffer nahmen in diesem Zeitraum im Zuge der umfangreichen Sondermaßnahmen weiter zu – bezogen auf den Anteil des harten Kernkapitals an den risikogewichteten Aktiva von 14 % auf 15,3 % (vgl. Abbildung 3) – womit Italien nah am EWU-Durchschnitt bleibt.

Dass das italienische Bankensystem trotz gewisser Schwächen weniger stark betroffen war als anfänglich befürchtet, liegt neben den umfangreichen staatlichen Hilfsmaßnahmen auch daran, dass es aufgrund zwischenzeitlicher Öffnungsphasen eine vergleichsweise starke wirtschaftliche Erholung gab und Italien schnell eine hohe Impfquote erreichte. Zudem trug auch die wirtschaftliche Zuversicht bei, die dadurch entstand, dass Italien umfangreiche EU-Gelder vor allem im Rahmen des EU-Aufbaufonds zugesagt wurden – und vor allem dass die breite Regierungskoalition unter dem neuen Ministerpräsidenten Draghi die Chance ergriff, einen umfangreichen Aufbau- und Resilienzplan vorzulegen (Matthes, 2021a).

Im Zuge der Coronakrise hat sich jedoch die enge Verflechtung zwischen Staat und Banken in Italien weiter verstärkt. Das gilt vor allem für die umfangreichen staatlichen Kreditgarantien, die eine beidseitige Abhängigkeit mit sich bringen. Zudem haben italienische Banken einen erheblichen Teil der erhöhten Staatsverschuldung in Form von weiteren Staatsanleihen aufgekauft, sodass der Anteil der heimischen

Abbildung 6

**Staat-Banken-Nexus: Heimische Staatsanleihen in Bankbilanzen – Entwicklung in Italien im Zeitverlauf**

Anteil der heimischen Staatsanleihen an der Bilanzsumme in %

Angaben für signifikante Banken unter EZB-Aufsicht.

Quellen: ECB (2022); Institut der deutschen Wirtschaft.

Staatsanleihen an der Bilanzsumme der italienischen Banken zwischenzeitlich von gut 10 % Ende 2019 auf knapp 12 % im August 2020 zunahm (vgl. Abbildung 6). Seitdem ging der Anteil bis November 2021 wieder auf 10,5 % zurück. Er bleibt damit aber der höchste aller Euroländer (vgl. Abbildung 7).

## Ausblick auf 2022 und potenzielle Risiken

Es ist zu erwarten, dass die bisher durch die staatlichen Hilfsmaßnahmen überdeckten Krisenprobleme stärker virulent werden, wenn Garantien und Moratorien allmählich abgebaut werden (IMF, 2021). Tatsächlich erwarten Fachleute für Italien einen überdurchschnittlichen Anstieg der Insolvenzen – sie dürften 2022 um etwa 15 % über dem Vorkrisenniveau von 2019 liegen (Lemmerle et al., 2021). Damit werden Kreditausfallraten und auch der Anteil notleidender Kredite wieder zunehmen. Zwar ist der erwartete Anstieg der Insolvenzen nicht außergewöhnlich hoch, doch ist es trotzdem geboten, die Situation von Banken und Unternehmen genau zu beobachten. Sollten die notleidenden Kredite nach der Coronakrise – etwa auch wegen höherer Zinsen – deutlich ansteigen, werden die italienischen Banken ihre Erfahrungen bei der Veräußerung solcher Kredite nutzen müssen.

Ein Anstieg der Kreditausfälle dürfte bei Auslaufen der Sondermaßnahmen auch bei der Privatkundschaft anstehen, wo die staatlichen Hilfen zu deutlich geringeren Ausfallraten beigetragen haben, wie Abbildung 5 zeigt. Doch sollten die Risiken hier nicht zu groß sein. Denn anders als in anderen Euroländern schlummern in Italien keine größeren Risiken im Immobiliensektor durch starke Anstiege bei Preisen und Hypotheken. Auch bei der Verschuldung der privaten Haushalte steht Italien passabel da. Sie stieg zwar in der Krise stark an, doch bleibt die Zinslast relativ zum verfügbaren Einkommen im Euroraumvergleich niedrig (ECB, 2021b). Stärker noch als in anderen Euroländern ist in Italien zudem der Anteil mit einer Zinsbindung von mehr als fünf Jahren

Abbildung 7

**Staat-Banken-Nexus: Heimische Staatsanleihen in Bankbilanzen – Vergleich der Euroländer**

Anteil der heimischen Staatsanleihen an der Bilanzsumme in %

Angaben für signifikante Banken unter EZB-Aufsicht.

Quellen: ECB (2022); Institut der deutschen Wirtschaft.

unter den Neukrediten an Haushalte gestiegen, was weniger verletzlich gegenüber möglichen Zinsanstiegen macht.

Das zentrale Risiko für das italienische Bankensystem verbirgt sich an anderer Stelle. Denn ein merklicher Zinsanstieg könnte mittelfristig die Tragfähigkeit der hohen italienischen Staatsschulden gefährden und damit indirekt auch die italienischen Banken. Aufgrund des sehr hohen Anteils der Staatsanleihen in den Bilanzen der italienischen Banken schlummert hier eine große Gefahr für die Zukunft. Denn mit steigenden Zinsen verlieren die Staatsanleihen an Wert. Da rund die Hälfte davon in Italien (2020) normalerweise nach Marktwert bilanziert werden muss (ECB, 2020b), würde dies kurzfristig zu Verlusten führen und das Kernkapital der italienischen Banken angreifen. Eine Simulation der EZB (ECB, 2020b) zeigt, dass sich Wertveränderungen von Staatsanleihen besonders stark auf italienische Banken auswirken verglichen mit dem Euroraumdurchschnitt. Wertminderungen bei Staatsanleihen können auch dadurch entstehen, dass EZB und Eurosystem weniger Staatsanleihen aufkaufen, wenn die krisenbedingten Anleihekaufprogramme wegen der gestiegenen Inflation beendet werden.

Während der Coronakrise hat die europäische Bankenaufsicht eine temporäre Ausnahme ermöglicht, um solche verlustbringenden Bewertungsänderungen weitgehend zu verhindern. Doch diese Sonderregel muss bei Auslaufen der Coronakrise wieder abgeschafft werden, um hinreichende Bilanzwahrheit zu ermöglichen. Dann würde die Verwundbarkeit des italienischen Bankensystems gegenüber Wertveränderungen der italienischen Staatsanleihen virulent – und es würde möglicherweise eine Kreditklemme drohen, die die italienische Wirtschaft in der Breite schwächt. Dies gilt umso mehr, als es mehrere weitere Transmissionskanäle zwischen Staatsanleihemärkten und den Finanzierungsbedingungen für Banken gibt (Demary und Matthes, 2013).

## Fazit und wirtschaftspolitische Einordnung

Die Altlasten der Euro-Schuldenkrise im italienischen Bankensystem mit Blick auf notleidende Kredite und Kapitalpuffer wurden recht weitgehend abgebaut, dies gilt aber nicht für den Nexus zwischen Staaten und Banken. Damit würde sich eine Krise bei den Staatsschulden direkt auf das Bankensystem und die italienische Wirtschaft übertragen. Dieser Befund hat weitreichende wirtschaftspolitische Implikationen. So erscheint es damit umso wichtiger, dass es bei der Reform des Stabilitäts- und Wachstumspakts nicht wie von Italien (und Frankreich) angestrebt (Draghi und Macron, 2022) zu einer Aufweichung der europäischen Fiskalregeln kommt. Zu wichtig ist der Erhalt der Tragfähigkeit der italienischen Staatsschulden, als dass sie durch neue Ausnahmen für nur eingeschränkt produktive öffentliche Investitionen gefährdet werden darf (Matthes, 2022).

Zudem lässt sich die Gefahr nicht vollständig ausschließen, dass Zinserhöhungen – über eine Spirale fehlenden Vertrauens und immer höherer Zinsen – letztlich zu einer Zahlungsunfähigkeit Italiens führen könnten. In einem solchen Extremfall wäre eine Umschuldung der Staatsschulden – auch im Sinne der No-Bailout-Regel – das Mittel der Wahl. Um diesen Schritt zu erleichtern, wurden im Zuge der Euro-Schuldenkrise sogenannte Umschuldungsklauseln in die Staatsanleihen der EWU-Länder aufgenommen, deren Handhabung in einem weiteren Reformschritt weiter vereinfacht wird (Matthes, 2021b). Doch macht der enge Staaten-Banken-Nexus in Italien eine Umschuldung politisch nahezu unmöglich, eben weil dies unweigerlich zu einer massiven Banken- und Wirtschaftskrise führen würde. Einer Umschuldung steht im Übrigen möglicherweise auch der hohe Anteil der Staatsanleihebestände von EZB und Eurosystem entgegen, weil damit bestehende Umschuldungsverfahren ausgehebelt zu werden drohen (Heinemann, 2021).

Diese Konstellation hat zweierlei politische Implikationen. *Zum einen* macht es ein solches Szenario der EZB schwerer, zur Inflationsbekämpfung die Zinsen zu erhöhen und aus den Staatsanleihekaufprogrammen konsequent auszusteigen – der Geldpolitik droht die fiskalische Dominanz. Vor allem die Veräußerung von Staatsanleihen aus ihren Anlagebeständen könnte zu Verwerfungen an den Finanzmärkten führen. Die Finanzstabilitätsrisiken auf den systemrelevanten Staatsanleihemärkten konkurrieren für die EZB daher zumindest implizit mit den Risiken einer Inflation. *Zum anderen* würde bei einer drohenden Zahlungsunfähigkeit der politische Druck enorm sein, Italien mit umfangreichen Rettungsprogrammen zu unterstützen. Es erscheint zumindest fraglich, ob sich die italienische Regierung auf die Reformen einlassen würde, die bei einem für diesen Fall angebrachten Hilfsprogramm des Europäischen Stabilitätsmechanismus unverzichtbar wären. Sollte dies nicht der Fall sein, würde ein Hilfsprogramm die No-Bailout-Klausel ad absurdum führen.

Schließlich spielt der Staaten-Banken-Nexus auch bei der geplanten Vollendung der Bankenunion eine wichtige Rolle, vor allem bei der avisierten Schaffung einer europäischen Einlagensicherung. Wenn die italienischen Banken bei Turbulenzen am Staatsanleihemarkt in Italien unter Druck gerieten, könnte es – wie schon zeitweise in der Euro-Schuldenkrise – zu einem Vertrauensverlust der italienischen Anlegenden in ihre Banken kommen – bis hin zu einem Bank Run. Eine europäische Einlagensicherung soll dem vorbeugen und Vertrauen schaffen. Doch wenn es aufgrund des Staaten-Banken-Nexus trotz einer solchen Hilfszusicherung zu Bankenkonkursen käme, müsste die europäische Einlagensicherung italienische Anlegende entschädigen. Diese Risikovergemeinschaftung könnte Banken in anderen Euroländern teuer zu stehen kommen (Demary, 2013). Daher war die mögliche Schaffung einer europäischen Einlagensicherung von Anfang an an den hinreichenden Abbau der Altlasten aus der Euro-Schuldenkrise gebunden.

Es ist damit richtig und unverzichtbar, dass der Koalitionsvertrag der neuen Bundesregierung zumindest indirekt einen Bezug zwischen der Schaffung einer Einlagen-Rückversicherung und dem Abbau des Staaten-Banken-Nexus herstellt. Dazu wäre es sinnvoll und nötig, die Bankenregulierung so zu ändern, dass Banken wie für andere Anlagen auch für Staatsanleihen ausreichende Risikovorsorge betreiben müssen, was bislang nicht der Fall ist (Schuster et al., 2013). Mit einer längeren Übergangsphase sollten Staatsanleihen daher nicht mehr als risikolos eingestuft werden, wie es bisher der Fall ist. Abbildung 7 zeigt, dass die (größeren) Banken in vielen anderen Euroländern mit einem deutlich geringeren Anteil von heimischen Staatsanleihen in ihren Bilanzen auskommen. Das sollte mittelfristig auch für Italien machbar sein. Dabei ist allerdings zu berücksichtigen, dass Banken wichtige Käuferinnen von Staatsanleihen sind, wenn Staaten diese am Primärmarkt zur Finanzierung herausgeben. Hier dürfen EZB und Eurosystem nicht agieren, weil das direkte Staatsfinanzierung wäre. Wenn die Banken – so die vermutliche Sorge in Italien – hier zurückhaltender wären, könnte dies zu Absatzproblemen und höheren Zinsen am Primärmarkt führen. Doch dieser Schluss greift zu kurz. Denn wenn die Staatsfinanzen als stabil gelten, werden auch andere Anlegende wie in- und ausländische Fonds und Versicherungen als Käufer am Primärmarkt auftreten. Eine Regierung hat es also selbst in der Hand, für eine glaubwürdige Fiskalpolitik zu sorgen. Der Stabilitäts- und Wachstumspakt kann ihr dabei als externer Glaubwürdigkeitsanker dienen und sollte nicht als einengendes Korsett gesehen werden.

**Title:** ***Italy's Banking System and the Coronavirus Crisis***

## References

[CR1] Demary, M. (2013), Ein Vorschlag für eine europäische Bankenunion ohne automatische Vergemeinschaftung von Bankverlusten, *IW Policy Paper*, 16.

[CR2] Demary, M. und J. Matthes (2013), Die EZB auf Abwegen? Teil 2: Sind die Staatsanleihekäufe eine Mandatsüberschreitung?, *IW Policy Paper*, 14.

[CR3] Draghi, M. und E. Macron (2021), The EU’s fiscal rules must be reformed, Opinion in Financial Times, 23. Dezember.

[CR4] EBA (2019), Risk Dashboard Annex — Credit Risk Parameters Q4 2019.

[CR5] EBA (2021), Risk Dashboard Annex — Credit Risk Parameters Q4 2021.

[CR6] EBF — European Banking Federation (2020), Banking Sector Performance 2019.

[CR7] ECB (2020a), Supervisory Banking Statistics, Fourth Quarter 2019.

[CR8] ECB (2020b), Financial Stability Review, November.

[CR9] ECB (2021a), Supervisory Banking Statistics, Third Quarter 2021.

[CR10] ECB (2021b), Financial Stability Review, November.

[CR11] ECB (2022), Statistical Data Warehouse, https://sdw.ecb.europa.eu/ (11. Januar 2022).

[CR12] Französischer Vorsitz im Rat der EU (2022), Aufschwung, Stärke, Zugehörigkeit — Das Programm der französischen EU-Ratspräsidentschaft.

[CR13] Lemmerle, M. et al. (2021), Insolvencies — We’ll be back, Alllianz/Euler-Hermes Resarch, Oktober.

[CR14] Heinemann, F. (2021), Die alte Maastricht-Ordnung steht auf dem Spiel, *Handelsblatt*, 9. November.

[CR15] IMF (2021), Article IV Staff Report Italy.

[CR16] Matthes, J. (2021a), Italiens Aufbauplan geht in die richtige Richtung, aber nicht weit genug, *IW-Kurzbericht*, 74, https://www.iwkoeln.de/fileadmin/user_upload/Studien/Kurzberichte/PDF/2021/IW-Kurzbericht_2021-Italiens-Aufbauplan.pdf (11. Januar 2022).

[CR17] Matthes J (2021). Reform des Europäischen Stabilitätsmechanismus — eine Einordnung. Wirtschaftsdienst.

[CR18] Matthes, J. (2022), Stabilität statt staatlicher Überforderung und strategischer Überdehnung — Konzept für eine effektive Reform des Stabilitäts- und Wachstumspaktes, *IW Policy Paper*, erscheint in Kürze.

[CR19] Schuster, T., F. Kövener und J. Matthes (2013), New Bank Equity Capital Rules in the European Union A Critical Evaluation, *IW Policy Paper*, 6.

[CR20] Sinn, H.-W. (2022), Target Salden (aktuelle Daten), https://www.hanswernersinn.de/de/themen/TargetSalden (11. Januar 2022).

[CR21] SPD, Bündnis 90/Die Grünen und FDP (2021), Mehr Fortschritt wagen — Bündnis für Freiheit, Gerechtigkeit und Nachhaltigkeit, Koalitionsvertrag.

